# ProSeeK: A web server for MLPA probe design

**DOI:** 10.1186/1471-2164-9-573

**Published:** 2008-11-28

**Authors:** Lorena Pantano, Lluís Armengol, Sergi Villatoro, Xavier Estivill

**Affiliations:** 1 Genes and Disease Program, Center for Genomic Regulation (CRG), Doctor Aiguader, 88, 08003 Barcelona, Catalonia, Spain

## Abstract

**Background:**

The technological evolution of platforms for detecting genome-wide copy number imbalances has allowed the discovery of an unexpected amount of human sequence that is variable in copy number among individuals. This type of human variation can make an important contribution to human diversity and disease susceptibility. Multiplex Ligation-dependent Probe Amplification (MLPA) is a targeted method to assess copy number differences for up to 40 genomic loci in one single experiment. Although specific MLPA assays can be ordered from MRC-Holland (the proprietary company of the MLPA technology), custom designs are also developed in many laboratories worldwide. After our own experience, an important drawback of custom MLPA assays is the time spent during the design of the specific oligonucleotides that are used as probes. Due to the large number of probes included in a single assay, a number of restrictions need to be met in order to maximize specificity and to increase success likelihood.

**Results:**

We have developed a web tool for facilitating and optimising custom probe design for MLPA experiments. The algorithm only requires the target sequence in FASTA format and a set of parameters, that are provided by the user according to each specific MLPA assay, to identify the best probes inside the given region.

**Conclusion:**

To our knowledge, this is the first available tool for optimizing custom probe design of MLPA assays. The ease-of-use and speed of the algorithm dramatically reduces the turn around time of probe design. ProSeeK will become a useful tool for all laboratories that are currently using MLPA in their research projects for CNV studies.

## Background

The technological evolution of platforms for assessing genome-wide copy number imbalances [1] has allowed the discovery of an unexpected amount of human sequence involved in duplications and deletions (termed copy number variants or CNVs). In terms of sequence coverage, this is the most important type of human variation identified so far and can make an important contribution to human diversity and disease susceptibility (see [2] for review). So far, derived from the study of several hundreds of individual genomes, ~19% of the euchromatic portion of the human genome has been reported as variable (mainly in copy number) [3]. Several studies have shown the relationship between CNVs and disease phenotypes [4,5].

**Figure 1:**
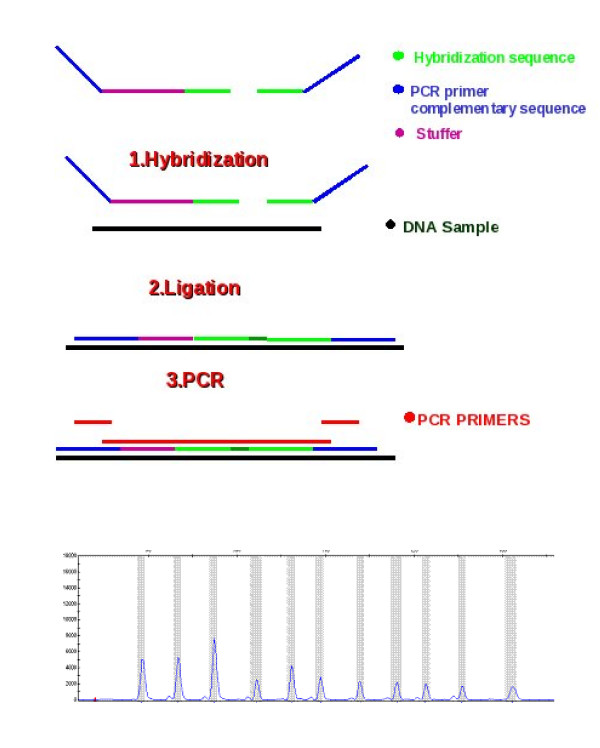
**MLPA assay**. Typical steps in a MLPA assay and the final output where each peak represents a probe in the experiment.

MLPA [6], Multiplex Ligation-dependent Probe Amplification, is a targeted method to assess copy-number differences for up to 40 genomic regions in one single experiment. Each MLPA probe is composed of two oligonucleotides that are only ligated, and subsequently amplified, if specifically hybridized to the target locus. The left probe oligonucleotide (LPO) is made of a complementary sequence of an universal forward PCR primer at its 5' end, plus the specific hybridizing sequence (LHS) at its 3' end. The right oligonucleotide (RPO) has the specific hybridizing sequence (RHS) at its 5' end followed by the complementary sequence to the reverse universal PCR primer, at the 3' end. After ligation all probes are amplified, by means of a universal primer pair, in a multiplex PCR reaction. This PCR produces loci-specific amplicons due to a stuffer sequence located between the hybridizing and the universal sequences. They are then resolved by capillary electrophoresis and copy number of each region is measured as a function of peak intensities of the MLPA amplification products (Figure 1).

Although specific MLPA assays can be ordered from MRC-Holland (the proprietary company of the MLPA technology), custom designs are also developed in many laboratories worldwide. After our own experience, an important drawback of custom MLPA assays is the time spent during the design of the specific oligonucleotides. Due to the large number of probes included in a single assay, a number of restrictions need to be met in order to maximize specificity and to increase success likelihood. Given the tedious stepwise procedure that is followed, the goal of ProSeeK is to automate the process of probe design and to obtain the best candidate probes for a given region.

## Implementation

ProSeeK is presented as an easy-to-use and point-and-click web interface. Is implemented in CGI (Common Gateway Interface) Perl scripts and made accessible to the user using PHP on top of an APACHE server with MYSQL database support. It is accessible through the Internet (at ) with IE5.0 and Netscape 7 or higher, from any platform. By making use of universally available web GUIs, the system solves the problem of portability of this software. No client-side software installation is required.

**Figure 2:**
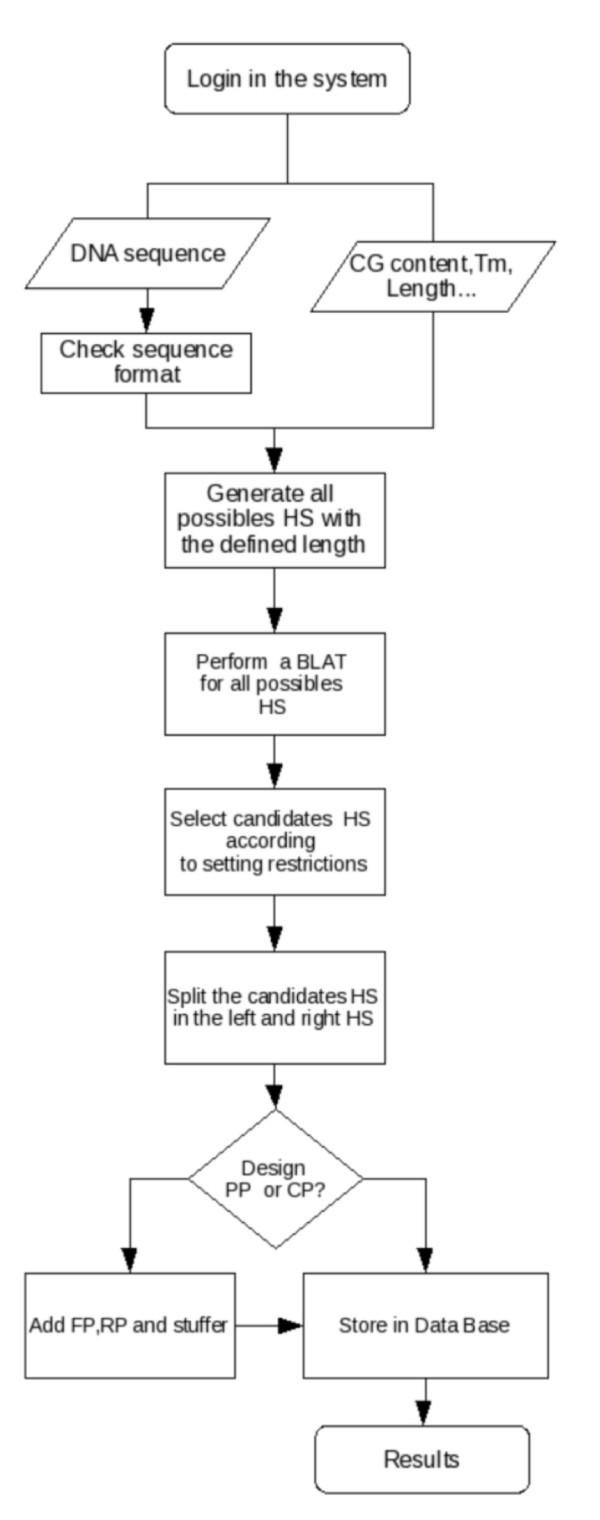
**Protocol**. Flowchart describing the implementation of ProSeek algorithm. HS (Hybridizing sequence), PP (Partial project), CP (complete project), FR (Forward primers), RP (Reverse primers).

The algorithm for probe design consists of several modules (Figure 2) that are run iteratively. (1) Sequence Checker, ensures that a valid sequence format is entered by the user. (2) Hybridization Finder, that identifies a set of hybridizing sequences (HSs), with the correct size, that are required to start and end with either a C or a G (according to the MRC-Holland protocol advises). Candidate HSs are filtered based on melting temperature and GC content, according to the set of thresholds provided by the user, and subsequently added primers and stuffers as needed. (3) Sequence Aligner, that performs a genome alignment using BLAT [7] to identify the optimal HS. Candidate HSs, regardless of having single or multiple matches to the genome, are filtered by the e-value of the alignment before passing to the next module. In the case that the HSs map onto a copy number variable (CNV) region or segmental duplication (SD) in the reference genome, the HSs are only recognized as optimal if the multiple matches are perfect and the other possible matches are below the e-value threshold. In the case that the probes designed are located in CNV or in SDs regions, this information is shown to the user in the output flagging them as 'CNR' and 'SD' respectively. (4) HS Trimmer, conveniently splits the HSs to fulfill user-entered criteria in terms of length, melting temperature and global sequence composition. (5) Results Generator, presents results to the user. ProSeeK can be asked to retrieve different results: the "partial" will only produce the optimal design for the left and right hybridizing sequences, while the "complete" will produce the whole oligonucleotide sequences corresponding to the LPO and RPO (see above). (6) Data Keeper, takes care of storing the results in a personal space of the database for future retrieval of the designs.

### Input to server

ProSeek requires the DNA sequence of the target region in which the MLPA probes will be designed. Several parameters can be used to restrict the probe design: (1) maximum GC content, (2) maximum melting temperature (Tm) of the hybridizing sequence, (3) Blat e-value (minimal length that the Blat will detect as a match), (4) hybridizing sequences (HS) length, (5) stuffer sequence, (6) sequence of the universal primers to flank the HS, and (7) desired probe length. (Additional file 1).

### Output from server

After computing all available possibilities, ProSeek produces a table in HTML format containing optimal probes which are presented to the user, together with their characteristics, which include position within the user-entered sequence, genome mapping, GC content, melting temperature, probe sequence, nucleotide length, self-folding capacity (i.e. DNA secondary structure prediction using DINAMelt Server [8]), and links to the UCSC Genome Browser [9] and to the Database of Genomic Variants [10] The projects are kept on the server for one month, so the users can retrieve their results at any time by returning to the website and identifying himself on the initial web page. (Additional file 1).

## Conclusion

A number of high-throughput technologies have become available to address the genome-wide detection of structural variations in humans. An important drawback of these new methods is that a huge amount of false positive results typically arise after analysis, thus it is mandatory to validate observations made with these technologies using alternative and more reliable approaches. Among others, due to its simplicity, robustness and relative low price, the MLPA is often used as a targeted method to assess copy-number differences. One important inconvenience is the required time for designing the probe-mixes to target the desired regions, since a lot of restriction should be fulfilled to get a sensitive, specific and reproducible experiment. To overcome this aspect, we developed ProSeek that produces the optimal probes for the regions of interest. ProSeeK is, to our knowledge, the first algorithm for the design of MLPA probes, that allows saving time and improving accuracy of MLPA assays.

## Availability and requirements

• Project name: ProSeeK

• Project home page: 

• Programming language: Perl

• License: GNU General Public License

## Authors' contributions

LP initiated this web server project, wrote the original source code, constructed the web interface, implemented it on the server and wrote the manuscript. LA conceived the server and participated in manuscript writing. XE revised and helped to write the manuscript. SV helped to design the web interface particularly from the viewpoint of an experimental research field. All authors contributed to the final manuscript and agreed the final version.

## Supplementary Material

Additional file 1 **Tutorial.** A complete tutorial explaining step by step the ProSeeK procedure. Click here for file
