# Supplementary material for: ProSeeK: A web server for MLPA probe design
Source: BMC Genomics. 2008 Nov 28;9:573. doi: 10.1186/1471-2164-9-573 (PMC2625369; doi:10.1186/1471-2164-9-573)
Supplement: Additional file 1 — Tutorial. A complete tutorial explaining step by step the ProSeeK procedure. [file 1471-2164-9-573-S1.pdf]

ProSeeK: A web server for MLPA probe design

Lorena Pantano[1], Lluis Armengol[1], Sergi Villatoro[1] and Xavier Estivill*[1]

[1]Genes and Disease Program, Center for Genomic Regulation (CRG), Doctor Aiguader, 88, 08003 Barcelona, Catalonia, Spain.

Email: LP - lorena.pantano@crg.es; LA - lluis.armengol@crg.es; SV - sergi.villatoro@crg.es; XE*- xavier.estivill@crg.es;

*Corresponding author

1 FAQs

1.1 What is MLPA?

Multiplex ligation-dependent probe amplification, is a targeted method to assess copy-number differences for up to 40 genomic regions in one single experiment. Each MLPA probe consists of two oligonucleotides. The left probe oligonucleotide (LPO) correspond to the complementary sequence of the universal forward PCR primer at its 5' end, and the specific hybridizing sequence (LHS) at its 3' end. The right probe (RPO) is made of the specific hybridizing sequence (RHS) at its 5' end followed by the complementary sequence of the reverse universal PCR primer at the 3' end. The probes hybridize to a specific nucleic acid sequence. Ligation only occurs at the specific site,and then all probes are amplified, by means of a universal primer pair, in a PCR reaction. This PCR produces amplicons of unique size, due to the stuffer sequence located between the hybridizing and the universal sequences, and are resolved by electrophoresis. The copy number of each region is reflected in the relative intensities of the MLPA probe amplification products.

1.2 What is ProSeeK?

ProSeeK helps you to design the best probes for a given MLPA assay.

1.3 How can I use ProSeeK?

ProSeeK only needs to be feed with the sequence of the region you are interested in (max 500 NTs).

1.4 What type of sequence can ProSeeK use?

ProSeeK needs a Fasta nucleotide sequence.

1.5 What type of design can you perform?

You can perform two type of designs:

- A complete mode of the probe where ProSeek designs a complete probe for a given sequence. In this case, you will obtain the probe with the left probe made of the forward primer, the stuffer if needed and the hybridizing sequence, and the right probe made of the reverse primer, the stuffer if needed and the hybridizing sequence.
- A partial mode where ProSeeK only desings the left and right hybridizing sequences.

1.6 What are the parameters?

- Cutoff CGs means the minimum CGs content in the probe.
- Cutoff CGs Right means the minimum CGs content in the right probe.
- Cutoff CGs Left means the minimum CGs content in the left probe.
- Cutoff Tm means the minimum Tm in the probe.
- Cutoff Tm Right means the minimum Tm in the right probe.
- Cutoff Tm Left means the minimum Tm in the left probe.
- MinScoreBlat refers to Blat sensitivity, that is the minimal length that the Blat will detect as a match in the Blat step.
- Min Length Probe means the minimum length for either the left or the right length probe.
- Max Length Probe means the maximum length for either the left or the right length probe.

1.7 What are the default parameters used by ProSeeK in the complete design mode?

- Forward Primer is the complementary sequence of the forward primer in the MLPA assay.
- Reverse Primer is the complementary sequence of the reverse primer in the MLPA assay.
- Stuffer is the sequence which ProSeeK uses to add at the left or right probes to get the desired length
- Length is the probe length you need

- Normalize size probes allows you to design both probes with same length.

1.8 Can I rescue my projects in any time?

Yes, ProSeeK stores all the projects in the database, each of them is associated to one user.

You can manage your projects at any time.

2 Tour Guide

2.1 Choose name project

Recommend to give an appropriate name which will identify all the set probes for one experiment, so then you will be able to recognize it easier in case you want to recover the results of the project. (Figure 1).

2.2 Simple design

In this mode, ProSeeK only designs the left and right hybridizing sequences. (Figure 2).

2.3 Complete design

In this mode ProSeek designs a complete probe for a given sequence. You will need to set the primers and the stuffer sequences.
(Figure 3).

2.4 Read Results

The results are given in a table format where different information is shown:

- Position inside the given sequence
- Position in the genome
- CGs content,Tm values and length
- Predicted fold of the probes
- Probe sequence included the primers or not depending on the selected parameters
- Links to UCSC and DataBase Genomic Variants Genome Browser

(Figure 4 and Figure 5).

Figures

Figure 1 - Step 1

First step: Introduce a name project.

Figure 2 - Step 2

Second step: Partial mode

Figure 3 - Step 3

Second step: Complete mode

Figure 4 - Step 3

Second step: Results from Partial design

Figure 5 - Step 4

Second step: Results from Complete design

Figure 1

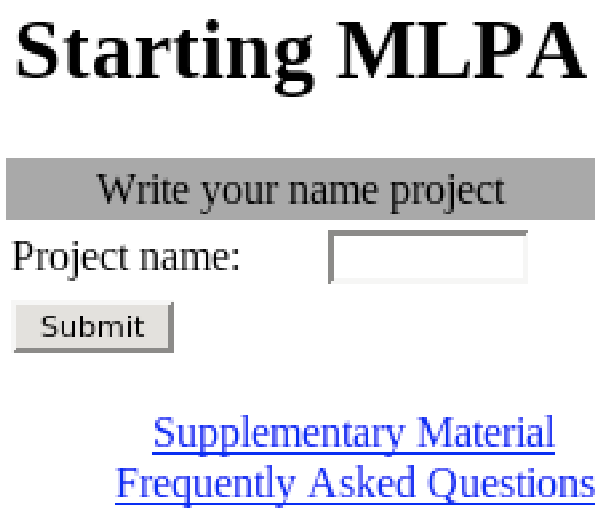
Starting MLPA
Write your name project
Project name:
Submit
Supplementary Material
Frequently Asked Questions

Figure 2

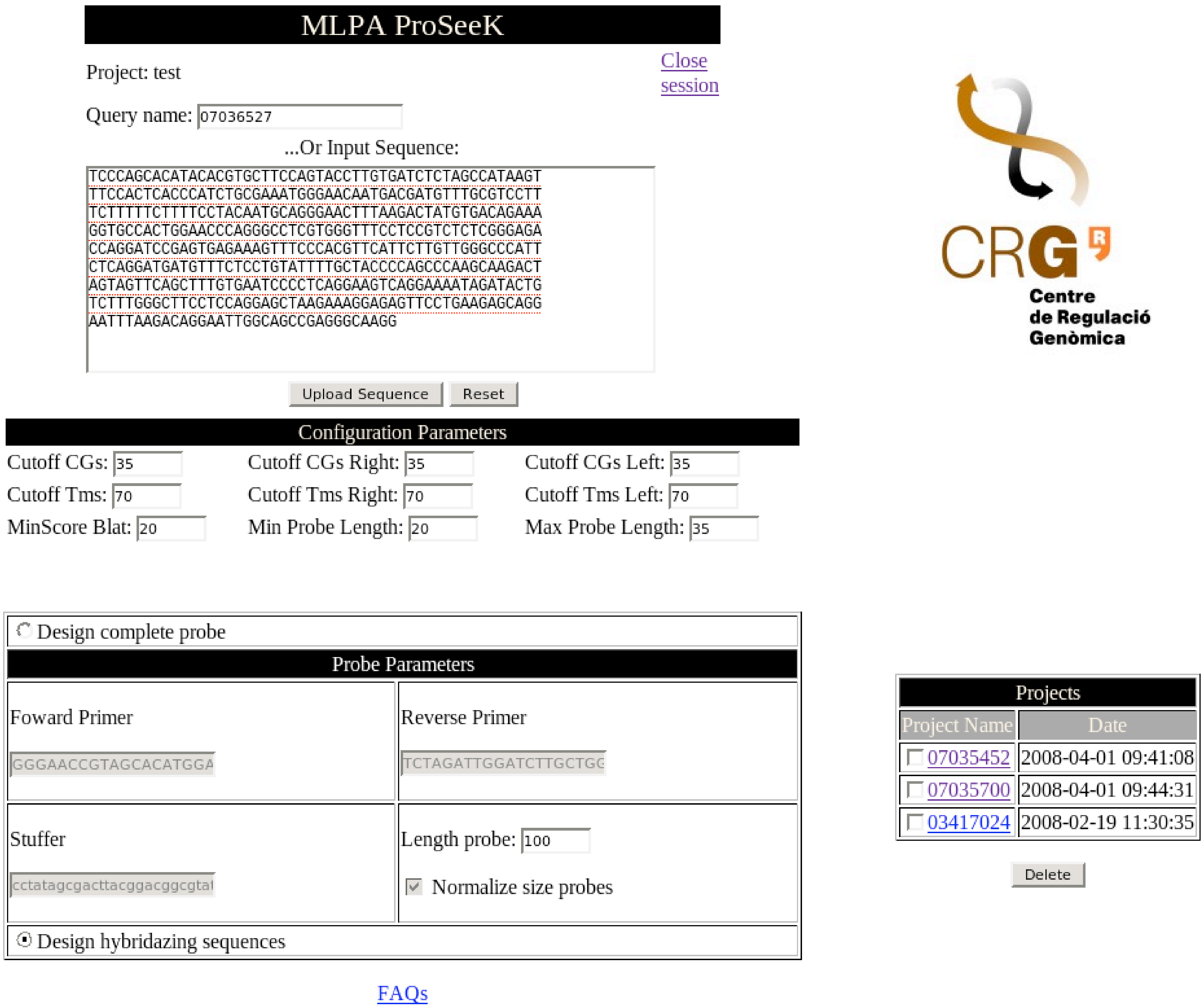

MLPA ProSeeK
Project: test
Close session
Query name: 07036527
...Or Input Sequence:
TCCCAGCACATACACGTGCTTCCAGTACCTTGTGATCTCTAGCCATAAGT
TTCCACTCACCCATCTGCGAAATGGGAACAATGACGATGTTTGCGTCCTT
TCTTTTTCTTTTCCTACAATGCAGGGAACTTTAAGACTATGTGACAGAAA
GGTGCCACTGGAACCCAGGGCCTCGTGGGTTTCCTCCGTCTCTCGGGAGA
CCAGGATCCGAGTGAGAAAGTTTCCCACGTTCATTCTTGTTGGGCCCATT
CTCAGGATGATGTTTCTCCTGTATTTTGCTACCCCAGCCCAAGCAAGACT
AGTAGTTCAGCTTTGTGAATCCCCTCAGGAAGTCAGGAAAATAGATACTG
TCTTTGGGCTTCCTCCAGGAGCTAAGAAAGGAGAGTTCCTGAAGAGCAGG
AATTTAAGACAGGAATTGGCAGCCGAGGGCAAGG
Upload Sequence
Reset
Configuration Parameters
Cutoff CGs: 35
Cutoff CGs Right: 35
Cutoff CGs Left: 35
Cutoff Tms: 70
Cutoff Tms Right: 70
Cutoff Tms Left: 70
MinScore Blat: 20
Min Probe Length: 20
Max Probe Length: 35
Design complete probe
Probe Parameters
Foward Primer
GGGAACCGTAGCACATGGA
Reverse Primer
TCTAGATTGGATCTTGCTGG
Stuffer
cctatagcgacttacggacggcgtat
Length probe: 100
Normalize size probes
Design hybridazing sequences
FAQs
CRG
Centre de Regulació Genòmica
Projects
Project Name
Date
07035452
2008-04-01 09:41:08
07035700
2008-04-01 09:44:31
03417024
2008-02-19 11:30:35
Delete

Figure 3

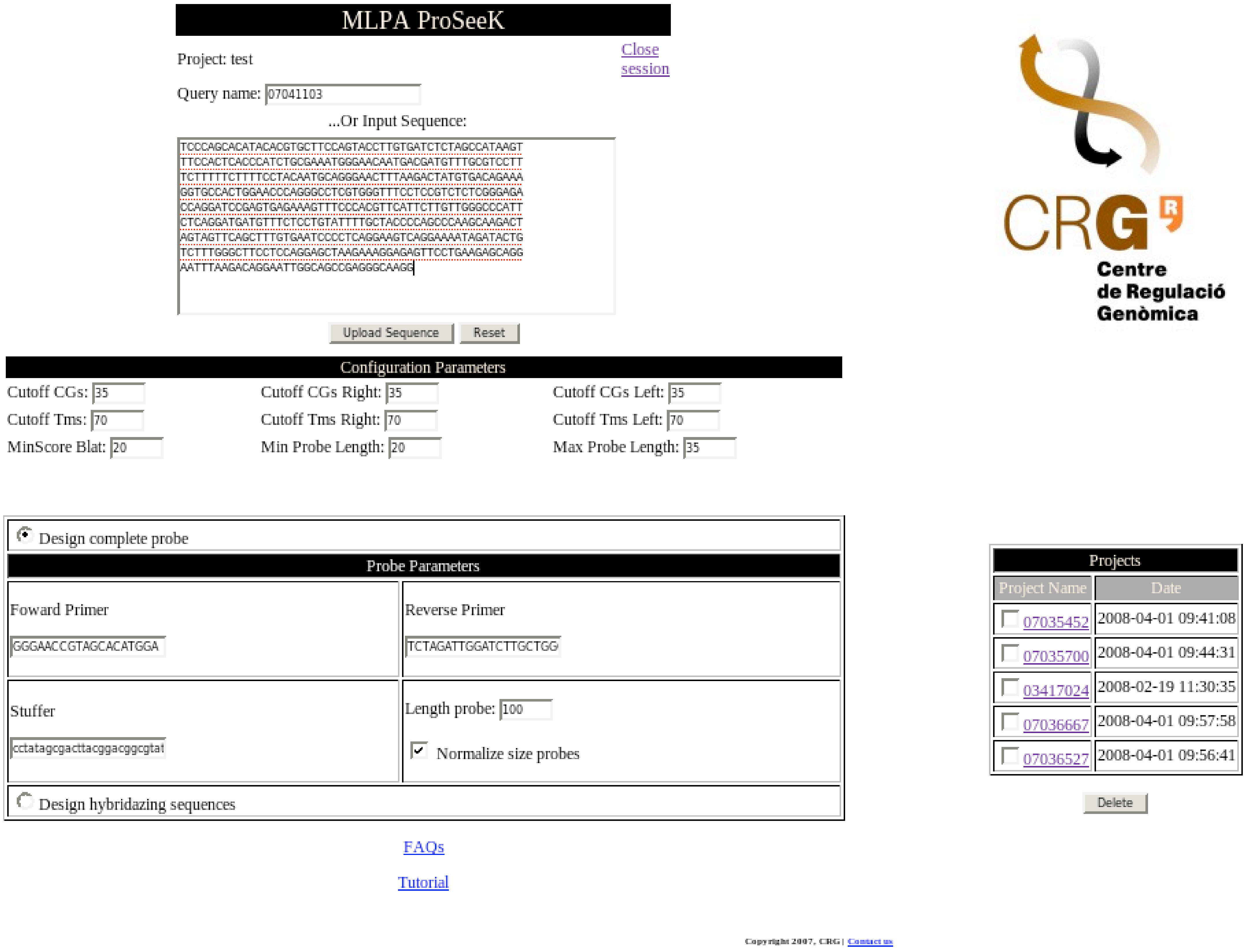
MLPA ProSeeK
Project: test
Close session
Query name: 07041103
...Or Input Sequence:
TCCCAGCACATACACGTGCTTCCAGTACCTTGTGATCTCTAGCCATAAGT
TTCCACTCACCCATCTGCGAAATGGGAACAATGACGATGTTTGCGTCCTT
TCTTTTTCTTTTCCTACAATGCAGGGAACTTTAAGACTATGTGACAGAAA
GGTGCCACTGGAACCCAGGGCCTCGTGGGTTTCCTCCGTCTCTCGGGAGA
CCAGGATCCGAGTGAGAAAGTTTCCCACGTTCATTCTTGTTGGGCCCATT
CTCAGGATGATGTTTCTCCTGTATTTTGCTACCCCAGCCCAAGCAAGACT
AGTAGTTCAGCTTTGTGAATCCCCTCAGGAAGTCAGGAAAATAGATACTG
TCTTTGGGCTTCCTCCAGGAGCTAAGAAAGGAGAGTTCCTGAAGAGCAGG
AATTTAAGACAGGAATTGGCAGCCGAGGGCAAGG
Upload Sequence
Reset
Configuration Parameters
Cutoff CGs: 35
Cutoff CGs Right: 35
Cutoff CGs Left: 35
Cutoff Tms: 70
Cutoff Tms Right: 70
Cutoff Tms Left: 70
MinScore Blat: 20
Min Probe Length: 20
Max Probe Length: 35
Design complete probe
Probe Parameters
Foward Primer
GGGAACCGTAGCACATGGA
Reverse Primer
TCTAGATTGGATCTTGCTGG
Stuffer
cctatagcgacttacggacggcgtat
Length probe: 100
Normalize size probes
Design hybridazing sequences
FAQs
Tutorial
Copyright 2007, CRG | Contact us
Centre de Regulació Genòmica
Projects
Project Name
Date
07035452 2008-04-01 09:41:08
07035700 2008-04-01 09:44:31
03417024 2008-02-19 11:30:35
07036667 2008-04-01 09:57:58
07036527 2008-04-01 09:56:41
Delete

Figure 4

ProSeeK Results

Sort by

HITs ▼ Sort

pos	chr	5'	3'	HITs	CGs_L	Tm_L	probe_L	length	Fold	CGs_R	Tm_R	probe_R	length	Fold	Link UCSC	Link DBGV
207	chr1	221980342	221980401	NUH-18	46.66	75.95	CCGAGTGAGAAAGTTTCCCACGTTCATTCT	30	Fold	48.38	77.90	TGTTGGGCCCATTCTCAGGATGATGTTTCTC	31	Fold	Genome Browser	Genome Browser
207	chr1	221980342	221980401	NUH-18	52.17	71.63	CCGAGTGAGAAAGTTTCCCACGT	23	Fold	45.16	77.45	TCATTCTTGTTGGGCCCATTCTCAGGATGAT	31	Fold	Genome Browser	Genome Browser
207	chr1	221980342	221980401	NUH-18	45.45	77.74	CCGAGTGAGAAAGTTTCCCACGTTCATTCTTGT	33	Fold	50	75.95	TGGGCCCATTCTCAGGATGATGTTTCTC	28	Fold	Genome Browser	Genome Browser
207	chr1	221980342	221980401	NUH-18	48.14	74.2	CCGAGTGAGAAAGTTTCCCACGTTCAT	27	Fold	47.05	79.13	TCTTGTTGGGCCCATTCTCAGGATGATGTTTCTC	34	Fold	Genome Browser	Genome Browser
107	chr1	221980242	221980299	NUH-18	38.70	72.22	CTTTTCCTACAATGCAGGGAACTTTAAGACT	31	Fold	53.57	78.61	ATGTGACAGAAAGGTGCCACTGGAACCC	28	Fold	Genome Browser	Genome Browser
107	chr1	221980242	221980299	NUH-18	37.5	71.82	CTTTTCCTACAATGCAGGGAACTTTAAGACTA	32	Fold	55.55	78.6	TGTGACAGAAAGGTGCCACTGGAACCC	27	Fold	Genome Browser	Genome Browser
207	chr1	221980342	221980401	NUH-18	50	74.12	CCGAGTGAGAAAGTTTCCCACGTTCA	26	Fold	45.71	79.17	TTCTTGTTGGGCCCATTCTCAGGATGATGTTTCTC	35	Fold	Genome Browser	Genome Browser
309	chr1	221980444	221980501	NUH-19	51.72	77.5	GCTTTGTGAATCCCCTCAGGAAGTCAGGA	29	Fold	43.33	73.22	AAATAGATACTGTCTTTGGGCTTCCTCCAG	30	Fold	Genome Browser	Genome Browser
309	chr1	221980444	221980501	NUH-19	45.45	77.83	GCTTTGTGAATCCCCTCAGGAAGTCAGGAAAAT	33	Fold	50	73.04	AGATACTGTCTTTGGGCTTCCTCCAG	26	Fold	Genome Browser	Genome Browser
309	chr1	221980444	221980501	NUH-19	50	77.68	GCTTTGTGAATCCCCTCAGGAAGTCAGGAA	30	Fold	44.82	72.97	AATAGATACTGTCTTTGGGCTTCCTCCAG	29	Fold	Genome Browser	Genome Browser
309	chr1	221980444	221980501	NUH-19	48.38	77.77	GCTTTGTGAATCCCCTCAGGAAGTCAGGAAA	31	Fold	46.42	72.69	ATAGATACTGTCTTTGGGCTTCCTCCAG	28	Fold	Genome Browser	Genome Browser
6	chr1	221980141	221980200	NUH-19	47.05	78.30	CACATACACGTGCTTCCAGTACCTTGTGATCTCT	34	Fold	48.14	73.87	AGCCATAAGTTTCCACTCACCCATCTG	27	Fold	Genome Browser	Genome Browser
6	chr1	221980141	221980200	NUH-19	50	71.25	CACATACACGTGCTTCCAGTACCT	24	Fold	45.71	77.93	TGTGATCTCTAGCCATAAGTTTCCACTCACCCATC	35	Fold	Genome Browser	Genome Browser
6	chr1	221980141	221980200	NUH-19	48.27	75.74	CACATACACGTGCTTCCAGTACCTTGTGA	29	Fold	46.87	76.38	TCTCTAGCCATAAGTTTCCACTCACCCATCTG	32	Fold	Genome Browser	Genome Browser
309	chr1	221980444	221980501	NUH-19	46.87	77.86	GCTTTGTGAATCCCCTCAGGAAGTCAGGAAAA	32	Fold	48.14	72.54	TAGATACTGTCTTTGGGCTTCCTCCAG	27	Fold	Genome Browser	Genome Browser

New Project

Figure 5

ProSeeK Results

Sort by

HITs Sort

pos	chr	5'	3'	HITs	CGs_L	Tm_L	probe_L	length	Fold	CGs_R	Tm_R	probe_R	length	Fold	Link UCSC	Link DBGV
207	chr1	221980342	221980397	NUH-17	48.14	74.2	GGGAACCGTAGCACATGGAcctatagCCGAGTGAGAAAGTTTCCCACGTTCAT	53	Fold	50	73.49	TCTTGTTGGGCCCATTCTCAGGATTCTAGATTGGATCTTGCTGGCAC	47	Fold	Genome Browser	Genome Browser
207	chr1	221980342	221980397	NUH-17	52.17	71.63	GGGAACCGTAGCACATGGAcctatagcgaCCGAGTGAGAAAGTTTCCCACGT	52	Fold	48	72.26	TCATTCTTGTTGGGCCCATTCTCAGTCTAGATTGGATCTTGCTGGCAC	48	Fold	Genome Browser	Genome Browser
207	chr1	221980342	221980397	NUH-17	50	74.12	GGGAACCGTAGCACATGGAcctatagCCGAGTGAGAAAGTTTCCCACGTTCA	52	Fold	48	73.77	TTCTTGTTGGGCCCATTCTCAGGATTCTAGATTGGATCTTGCTGGCAC	48	Fold	Genome Browser	Genome Browser

New Project
